# Management of COPD and comorbidities in COPD patients by dispensing pharmaceutical care following Global Initiative for Chronic Obstructive Lung Disease-guidelines (GOLD guidelines 2020): a prospective randomized clinical trial

**DOI:** 10.1186/s12913-026-14802-w

**Published:** 2026-06-16

**Authors:** Hafsa Kanwal, Gaber E. Eldesoky, Umm-e- Kalsoom, Saima Mushtaq, A. A. Haral, Yu Fang, Amjad Khan

**Affiliations:** 1 Department of Pharmacy, Quaid-i-Azam University, Islamabad, Pakistan; 2 Department of Chemistry, College of Science, King Saud University, Riyadh, Saudi Arabia; 3 Department of Pharmacy, The First Affiliated Hospital, Xi’an Jiaotong University, Xi’an, China; 4 Department of Pharmacy Administration and Clinical Pharmacy, School of Pharmacy, Health Science Center, Xi’an Jiaotong University, Xi’an, China; 5 Department of Chemistry, The University of Lahore, Lahore, Pakistan

**Keywords:** COPD, GOLD guidelines, Pharmaceutical care, COPD management, Role of pharmacist in disease management, QoL (Quality of Life)

## Abstract

**Background:**

Chronic Obstructive Pulmonary Disease (COPD) is a major global health burden, driving high rates of morbidity, mortality, and economic strain. Its prevalence is rising, especially among aging populations and in areas with heavy tobacco and pollutant exposure. Frequent comorbidities like cardiovascular disease and diabetes complicate care, necessitating multidisciplinary approaches beyond standard pharmacotherapy.

**Methods and findings:**

This prospective randomized clinical trial investigated the impact of individualized care aligned with Global initiative for chronic obstructive Lungs disease (GOLD guidelines)—on clinical outcomes in COPD patients with comorbidities. 120 patients were randomly allocated to three arms: standard care, pharmacist counselling, and comprehensive pharmaceutical care. Participants were followed for 24 weeks, and outcome measures included assessments of symptom severity (via CAT and mMRC scores), lung function (FEV₁), exacerbation frequency, and quality of life. Arm 2 (Pharmaceutical Care) significantly lowered CAT scores to 18.82 ± 13.69 compared to 29.88 ± 13.69 in the Arm 0 (control group) (p < 0.001) and reduced mMRC ratings to 0.91 ± 0.39 versus 1.36 ± 0.39 (p < 0.001). Arm 2 experienced the smallest FEV₁ (% predicted) change (–2.76 ± 2.52) versus –9.73 ± 2.52 in the control group, with a post-intervention mean difference of –9.70 ± 2.83 (p = 0.001). The duration of moderate exacerbations was significantly shorter in Arm 2 (1.50 ± 1.49 weeks) compared to 3.23 ± 1.49 weeks in controls (p < 0.001). Disease progression scores were lower in Arm 2 (1.15 ± 0.36) versus 1.45 ± 0.44 in the Arm 0 (p = 0.016). Quality of life significantly improved in the Arm 2 compared to Arm 0 and Arm 1, as reflected by lower SGRQ scores (p < 0.001).

**Conclusions:**

In conclusion, implementing pharmaceutical care based on GOLD 2020 guidelines not only enhances symptom management and preserves lung function but also significantly improves quality of life in COPD patients. This study underscores the critical role of pharmacists in multidisciplinary care teams, focusing the patient education and individualized patient care, especially in resource-limited settings.

**Clinical trial registration:**

This clinical trial has been registered in ANZCTR clinical trials registry: **ACTRN12622000234718** (https://www.anzctr.org.au/ACTRN12622000234718.aspx).

**Trial registration:**

This clinical trial has been registered in **Australian New Zealand Clinical Trials** **Registry: Trial ID (ACTRN12622000234718)**, Date of registration **09/02/2022** and updated at **02/03/2023**.

**Trial Protocols:**

These protocols are the detailed version of the registered clinical trial that has been published in **Heliyon** 10.1016/j.heliyon.2023.e21539.

**Supplementary Information:**

The online version contains supplementary material available at 10.1186/s12913-026-14802-w.

## Highlights

The findings of this study underscore the significant impact of pharmaceutical care on the management of COPD. A structured pharmaceutical intervention led to a notable reduction in CAT scores, demonstrating an improvement in symptom control. Patients receiving pharmaceutical care exhibited a significant decrease in CAT scores Furthermore, the decline in FEV₁ (% predicted), a key indicator of lung function deterioration, was minimized in the pharmaceutical care group, with a significantly lower reduction compared to the control group, suggesting a protective effect against disease progression.

Quality of life enhancements were most pronounced among patients receiving pharmaceutical care. This highlights the role of comprehensive care in enhancing daily functioning and overall well-being.

Lastly, the study found that pharmaceutical care contributed to a slower disease progression compared to standard care, as reflected in significantly lower disease progression scores. These findings collectively highlight the critical role of pharmaceutical care in optimizing COPD management, improving patient outcomes, and reinforcing the importance of integrating pharmacists into multidisciplinary healthcare teams for chronic disease management.

**Supplementary Information:**

The online version contains supplementary material available at 10.1186/s12913-026-14802-w.

## Introduction

Chronic obstructive pulmonary disease (COPD) is one of the leading causes of morbidity and mortality [1–3] worldwide, representing a significant public health challenge due to its progressive nature and the complex management it demands [4]. Characterized by persistent airflow limitation and a chronic inflammatory response in the airways, COPD not only affects pulmonary function but also imposes a heavy socioeconomic burden on patients, healthcare systems, and society at large [5–9]. Recent estimates indicate that the prevalence of COPD continues to rise, particularly in aging populations and in regions with high exposure to risk factors such as tobacco smoke, occupational pollutants, and environmental contaminants [10–12].

Despite significant advances in the understanding of COPD pathophysiology and the development of novel therapeutic agents, effective management remains challenging [6, 13, 14]. The complexity of treatment strategies including both pharmacologic and nonpharmacologic interventions requires a multifaceted approach [15–18]. This is particularly true as COPD patients often present with multiple comorbidities that further complicate disease management and treatment adherence [19, 20]. In this context, the role of pharmaceutical care has garnered increasing attention as an essential component of comprehensive COPD management [21, 22]. Pharmaceutical care, with its patient-centered focus, not only ensures the appropriate use of medications but also plays a crucial role in educating patients, monitoring drug efficacy, and mitigating adverse effects [23–25].

The Global Initiative for Chronic Obstructive Lung Disease (GOLD) guidelines serve as the cornerstone for evidence-based COPD management [26, 27]. The GOLD 2020 report provides a comprehensive framework for the diagnosis, treatment, and long-term management of COPD, emphasizing the importance of individualized care plans based on disease severity and patient-specific factors [28–30]. While this study follows the GOLD 2020 framework, it is important to note that subsequent GOLD updates have introduced modifications to patient grouping and management pathways. However, the present clinical trial was designed, registered, and initiated during the period when the GOLD 2020 report represented the prevailing evidence-based standard, and therefore the study design and patient stratification were aligned with that guideline version. Among its key recommendations, the guidelines advocate for a multidisciplinary approach that integrates the expertise of healthcare professionals across different specialties, including pharmacists [31–33]. This collaborative model is designed to optimize therapeutic outcomes, improve quality of life, and reduce the incidence of acute exacerbations—a primary driver of COPD-related hospitalizations and healthcare costs [34, 35]. Furthermore, based on the baseline characteristics of the cohort, including higher CAT scores, mMRC ratings, and exacerbation history, most participants would likely correspond to higher symptom-burden categories under updated GOLD classifications.

Patient counselling in medication management, are uniquely positioned to contribute to the implementation of GOLD guidelines in routine clinical practice [36, 37]. The concept of dispensing pharmaceutical care goes beyond mere medication distribution; it involves comprehensive patient counselling, regular monitoring of treatment adherence, and ongoing assessment of therapeutic effectiveness [37–39]. Recent studies have demonstrated that pharmacist-led interventions can significantly improve clinical outcomes in COPD patients by reducing exacerbation rates, enhancing medication adherence, and ultimately lowering healthcare utilization [40, 41]. Such interventions are particularly crucial in settings where patients may have limited access to specialized respiratory care, making the role of community and hospital pharmacists even more vital [42].

However, despite the promising evidence supporting pharmacist involvement in COPD management, there remains a paucity of robust clinical trials specifically investigating the impact of dispensing pharmaceutical care guided by the GOLD 2020 recommendations [39, 43]. The majority of existing literature focuses on isolated pharmaceutical interventions or non-randomized observational studies, which limits the generalizability of their findings. Moreover, the heterogeneity of patient populations and variations in healthcare delivery models across different regions further complicate the assessment of such interventions’ efficacy [44, 45]. Hence, a well-designed prospective randomized clinical trial is needed to rigorously evaluate the clinical and outcomes associated with this comprehensive pharmaceutical care model [46, 47].

The present study was conceived to address this critical gap by investigating the management of COPD patients through the dispensing of pharmaceutical care in accordance with the GOLD guidelines. By employing a prospective randomized clinical trial design, the study aims to provide high-quality evidence on how structured pharmaceutical interventions can enhance clinical outcomes in COPD. In particular, the aim of the study is the implementation the recommendations of the GOLD guidelines in healthcare settings in Pakistan whether a pharmacist-led care model can improve medication adherence, reduce the frequency and severity of exacerbations, and ultimately lead to better overall disease management compared to standard care practices.

### Objectives

1. Research hypothesis: integrating the standardized protocols outlined in the GOLD guidelines into clinical practices will enhance COPD management in Pakistan.
2. Study objectives: the objective of this research is to evaluate the enhancement of COPD management by implementing standardized treatment guidelines, to improve treatment outcomes, slow down disease progression and enhancing the quality of life. This endeavor is expected to have a positive impact by reducing the frequency of acute exacerbations, emergency visits, and hospital stays.

## Materials and methods

### Study design

This study is a prospective, exploratory, randomized controlled clinical trial designed to evaluate the impact of integrating standardized GOLD-guidelines into routine COPD care. The trial employed a three‐arm design with an equal 1:1:1 allocation ratio. Participants were randomly assigned to one of the following groups: Arm 0, Arm 1 and Arm 2 (Pharmaceutical Care). The design adheres to the Standard Protocol Items: Recommendations for Interventional Trials (SPIRIT) guidelines to ensure transparency and reproducibility.

### Randomization

Participants were randomly allocated to the three study arms using a computer-generated randomization sequence, ensuring equal distribution of participants among the intervention groups.

### Sample size calculation

The sample size was calculated based on the primary clinical outcome of change in CAT score between study groups after intervention. Assuming a moderate effect size corresponding to an expected mean difference of approximately 5 points in CAT score with an estimated standard deviation of 6 points, a two-sided α level of 0.05, and 80% statistical power, the minimum required sample size was estimated for comparison across the three study arms. To compensate for potential attrition during follow-up, additional participants were recruited.

### Quality of life measurement

Quality of life was assessed using the St. George’s Respiratory Questionnaire (SGRQ), a validated disease-specific instrument widely used to measure health-related quality of life in patients with COPD.

### Spirometry

Spirometry was performed according to standardized international guidelines. FEV₁ (% predicted) values were calculated using race-neutral reference equations, in accordance with current recommendations to avoid race-based bias in lung function assessment. Disease severity was classified based on the GOLD 2020 criteria using post-bronchodilator FEV₁ (% predicted) values.

### Blinding

Due to the nature of the interventions (education and pharmaceutical care), blinding of participants and investigators was not feasible, and the study was conducted as an open-label trial.

### Recruitment and intervention timeline

Patient recruitment was conducted between [September 2021 to October 2022], and participants were followed for 24 weeks follow-up duration to evaluate clinical outcomes.

### Study setting and population

The study was conducted at a tertiary care hospital, which provides specialized respiratory care and serves a diverse patient population. Although the study was conducted in a single center, the findings may be generalizable to similar tertiary healthcare settings in the country. The trial was conducted at the Medical Outpatient Department of Holy Family Hospital, Rawalpindi Medical University, Pakistan.

### Eligibility criteria

#### Diagnostic criteria

Participants having a clinical diagnosis of COPD confirmed by spirometry—a post-bronchodilator Forced Expiratory Volume in 1 s (FEV₁) to Forced Vital Capacity (FVC) ratio of less than 0.70. The target population includes patients presenting with dyspnea and chronic productive cough.

#### Inclusion criteria

Eligible participants had met the following criteria at the time of randomization: Receiving care exclusively in the outpatient COPD clinics, confirmed COPD diagnosis by a hospital consultant (FEV₁/FVC < 0.70 with a history of at least two acute exacerbations or emergency visits in the past 12 months), ability to provide informed consent after a comprehensive explanation of the study procedures and willingness to comply with scheduled follow-up assessments and interventions.

#### Exclusion criteria

Participants were excluded if they had severe mobility limitations preventing clinic attendance, inability to reliably complete follow-up visits, terminal comorbid illnesses, or clinically diagnosed congestive heart failure (either systolic or diastolic). Patients currently participating in pulmonary rehabilitation or those who had previously received structured COPD management consultation from a clinical pharmacist or pulmonary physiotherapist were also excluded.

### Intervention

The study interventions were delivered according to predefined structured protocols developed based on the GOLD 2020 COPD management guidelines. Participants were allocated to three study arms: usual care (Arm 0), educational intervention (Arm 1), and pharmacist-led pharmaceutical care (Arm 2) Pharmaceutical Care. While a brief description of each intervention is presented below, the detailed intervention protocols and workflow diagrams are provided in Supplementary File S1.

#### Arm 0 (usual care)

Participants in this group were continued with their routine treatment as prescribed by their primary physicians without any additional intervention from the study team.

#### Arm 1 (educational intervention)

Participants received a single, 30-minute face-to-face counselling session delivered by the principal investigator. The complete educational intervention protocol, including the components of patient education and follow-up reinforcement procedures, is provided in Supplementary File S1.

#### Arm 2 (pharmaceutical care)

In addition to the educational intervention provided in Arm 1, participants in Arm 2 (Pharmaceutical Care) have received:

1. **Standard Diagnostic Assessment**: Using recommended procedures to confirm COPD diagnosis and to classify patients according to GOLD stages.
2. **Pharmacological Management**: Participants in Arm 2 (Pharmaceutical Care) received a structured pharmaceutical care intervention delivered by a clinical pharmacist in collaboration of physician in addition to the educational intervention described for Arm 1. The intervention was guided by the GOLD 2020 treatment algorithm (Table 1) and included individualized pharmacotherapy optimization, inhaler technique training, adherence monitoring, and comorbidity management. Pharmacological therapy was reviewed at each follow-up visit and adjusted when necessary according to symptom severity (CAT and mMRC scores), exacerbation history, and spirometry results.
3. **Monitoring of Co-morbidities**: COPD-associated comorbidities, including hypertension, diabetes mellitus, cardiovascular diseases, gastroesophageal reflux disease (GERD), anxiety/depression, and respiratory infections. Monitoring was conducted using a standardized follow-up protocol applied uniformly across participants in the pharmaceutical care arm. Assessment methods included review of clinical history, medication profiles, physical examination findings, routine laboratory investigations (where available), spirometry results, exacerbation history, blood pressure and glycemic status records. Participants identified with uncontrolled comorbidities or medication-related issues were referred to the treating physician for further therapeutic evaluation and optimization according to GOLD 2020 recommendations.
4. The detailed pharmaceutical care intervention protocol, including medication review procedures, pharmacotherapy optimization, inhaler technique assessment, adherence monitoring, and comorbidity monitoring, is described in Supplementary File S1.

**Table 1:** COPD pharmacological treatment algorithm according to GOLD 2020 guidelines

	Appropriateness		Inappropriateness	
Group	First choice	Alternative choice	Under-treatment	Over-treatment
A	A bronchodilator	Change the bronchodilator	No bronchodilator prescribed	LABA+LAMA, ICS or any combination treatment
B	LAMA or LABA	LAMA+LABA	Only short-acting bronchodilator	LAMA+LABA + ICS, ICS+LABA, ICS+LAMA
C	LAMA	LAMA+LABA or ICS+LABA	Only ICS or LABA or SABA	ICS+LAMA, ICS+LABA+LAMA
D	LAMA+LABA or LAMA or ICS+LABA	If still exacerbation present ICS+LABA+LAMA	Only ICS or SABA or LABA, ICS+ LAMA	

*SABA –* Short-acting β₂-agonist, *LABA* – Long-acting β₂-agonist, *SAMA* – Short-acting muscarinic antagonist, *LAMA* – Long-acting muscarinic antagonist, *ICS* – Inhaled corticosteroid

### Outcome measures

1. **Quality of Life**: Measured using the St. George’s Respiratory Questionnaire at baseline and after 24 weeks.
2. **Acute Exacerbations/Hospital Visits**: The number of emergency visits and hospital admissions recorded from patient medical records over the 6-month period.
3. **Disease Progression**: Assessed by spirometry (FEV₁ (% predicted) measurements) performed before and after the intervention.

A pictorial representation of the intervention workflow is provided in Supplementary Figure S1.

### Statistical analysis

Continuous variables were assessed for normality using the Shapiro–Wilk test and visual inspection of Q–Q plots. Normally distributed variables are presented as mean ± standard deviation (SD), while skewed variables are reported as median with interquartile range (p25–p75). Categorical variables are presented as frequencies and percentages.

Baseline characteristics and clinical outcomes were compared across the three study arms (usual care, educational intervention, and pharmaceutical care) using one-way analysis of variance (ANOVA) for normally distributed variables and the Kruskal–Wallis test for non-normally distributed variables. Homogeneity of variances was evaluated using Levene’s test. When significant differences were observed, to control for multiple comparisons, Tukey’s HSD post-hoc correction was applied for pairwise group comparisons. Categorical variables were analyzed using the Chi-square test or Fisher’s exact test when appropriate. Missing data were minimal and handled using a complete-case analysis approach. A two-sided p-value < 0.05 was considered statistically significant [48]. To account for baseline differences in lung function, post-intervention outcomes were analyzed using analysis of covariance (ANCOVA) with baseline FEV₁ included as a covariate.

### Ethical considerations

The study protocol has been reviewed and approved by the institutional review boards (IRBs) under reference No. #BEC-FBS-QAU2021-269. Written informed consent was obtained from all participants prior to enrolment. Participants were informed of their right to withdraw at any time without affecting their standard care. Data confidentiality was rigorously maintained through anonymization and restricted access, with only the principal investigator having access to identifiable information until publication. All study procedures involving human participants were conducted in accordance with the ethical principles of the Declaration of Helsinki and relevant institutional guidelines and regulations.

## Results

**Figure:**
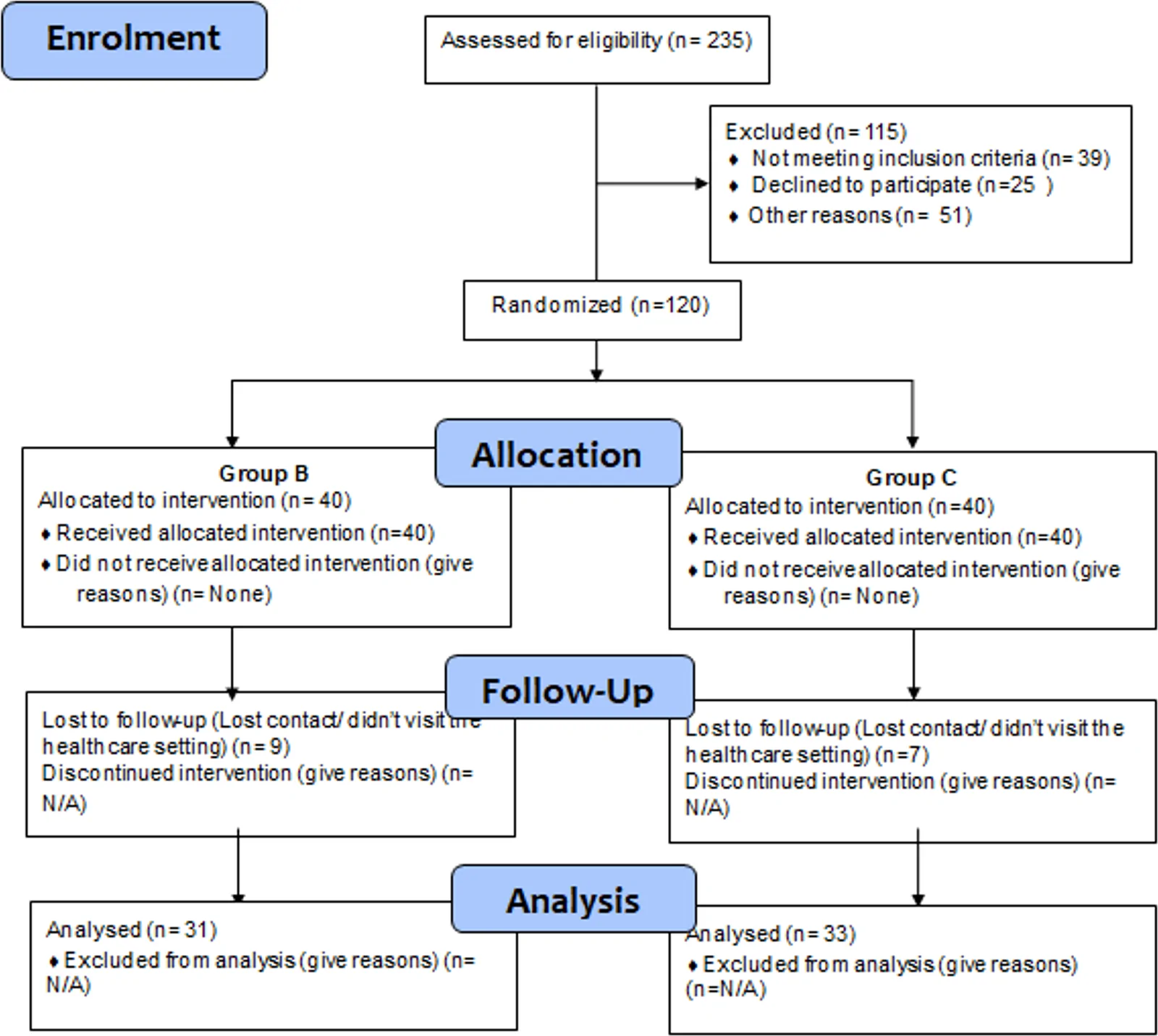

No major protocol deviations or cross-over between study arms were observed during the follow-up period, and all participants received their assigned interventions as per protocol.

A total of 97 COPD patients were included in the study and randomly allocated to the Arm 0 (*n* = 33), Arm 1 (*n* = 31), and Arm 2 (*n* = 33) groups. The mean age, sex distribution, and body mass index were comparable across the three groups.

Most participants were classified as GOLD stage II, followed by stage III. According to the GOLD 2020 ABCD classification, the majority of patients belonged to Group B, while smaller proportions were categorized in Group D. Baseline symptom burden assessed by CAT score and mMRC dyspnea scale was also recorded for all participants as presented in Table 2.

**Table 2:** Demographics of the patients

Variable	Arm 0 (*n* = 33)	Arm 1 (*n* = 31)	Arm 2 (*n* = 33)
**Age (years)**,** mean ± SD**	45.36 ± 18.36	43.48 ± 20.39	36.82 ± 14.36
**Male**,** n (%)**	16 (48.5%)	15 (48.4%)	17 (51.5%)
**Female**,** n (%)**	17 (51.5%)	16 (51.6%)	16 (48.5%)
**Body Mass Index (kg/m²)**	23.12 ± 5.35	24.87 ± 5.91	24.57 ± 4.24
**CAT score**	29.88 ± 2.19	24.84 ± 0.86	18.82 ± 5.89
**mMRC score**	1.36 ± 0.49	1.00 ± 0.26	0.91 ± 0.38
**GOLD Stage**			
GOLD 1	1 (3.0%)	4 (12.9%)	6 (18.2%)
GOLD 2	19 (57.6%)	23 (74.2%)	24 (72.7%)
GOLD 3	13 (39.4%)	4 (12.9%)	3 (9.1%)
GOLD 4	0	0	0
**GOLD Group**			
Group A	0	0	2 (6.1%)
Group B	26 (78.8%)	26 (83.9%)	30 (90.9%)
Arm 2 (Pharmaceutical Care)	0	0	0
Group D	7 (21.2%)	5 (16.1%)	1 (3.0%)

The Table 3 provides a comprehensive summary of the baseline characteristics, clinical measurements, imaging assessments, and treatment details of the study sample. additional baseline demographic and clinical characteristics of participants across the three study arms. Anthropometric measures, spirometric parameters, and blood eosinophil levels were comparable among the usual care, educational intervention, and pharmaceutical care groups. Baseline demographic and clinical characteristics were generally comparable across the three study arms prior to intervention. These findings indicate that the study groups were well balanced at baseline prior to the intervention.

**Table 3:** Health related variables at baseline

Variable	Arm 0 (*n* = 33)	Arm1(*n* = 31)	Arm2 (*n* = 33)
**Anthropometric Characteristics**			
Height (cm)	161.9 ± 10.4	162.8 ± 10.6	162.6 ± 10.7
Weight (kg)	63.1 ± 14.6	63.9 ± 14.9	63.6 ± 14.8
**Spirometry**			
FEV₁pre	72.4 ± 11.0	73.1 ± 10.8	73.0 ± 11.2
FEV₁/FVC	81.0 ± 2.1	81.3 ± 2.2	81.2 ± 2.1
Blood eosinophils (10⁹/L)	3.15 ± 0.84	3.21 ± 0.86	3.23 ± 0.85
**Respiratory Assessment**			
CAT score	24.8 ± 5.9	24.2 ± 5.7	24.5 ± 6.0
**mMRC Dyspnea Rating**			
No dyspnea except strenuous exercise	2 (6.1)	1 (3.2)	2 (6.1)
Shortness of breath walking uphill	26 (78.8)	25 (80.6)	26 (78.8)
Dyspnea limits walking pace	5 (15.1)	5 (16.1)	5 (15.1)
**Exacerbation History**			
Moderate exacerbations	**2 (1–3)**	**2 (1–2)**	**2 (1–2)**
Duration of moderate exacerbation (weeks)	**2 (1–4)**	**2 (1–3)**	**2 (1–3)**
Severe exacerbations	**0 (0–1)**	**0 (0–1)**	**0 (0–1)**
Duration of severe exacerbation (weeks)	**1 (1–2)**	**1 (1–2)**	**1 (1–2)**

### Symptom control (CAT and mMRC)

Post-intervention outcomes were compared across the three study groups after adjusting for baseline FEV₁ using analysis of covariance (ANCOVA). A statistically significant difference was observed between groups for symptom scores and quality of life measures. The pharmaceutical care group demonstrated significantly lower CAT scores and mMRC dyspnea ratings compared with usual care (*p* < 0.001), indicating improved symptom control. Similarly, quality of life, as assessed by SGRQ scores, was significantly better in Arm 2 compared with both usual care and educational intervention groups (*p* < 0.001) (Table 4).

### Lung function (FEV₁)

In terms of lung function, the pharmaceutical care group showed higher post-intervention FEV₁ (% predicted) values compared with the usual care group, suggesting a protective effect against decline in lung function (*p* = 0.001). However, no statistically significant differences were observed between groups for the number of moderate or severe exacerbations (*p* > 0.05), although a trend toward fewer exacerbations was noted in the Arm 2. Overall, these findings indicate that pharmaceutical care provides significant improvements in symptom burden, quality of life, and preservation of lung function compared with usual care (Table 4).

**Table 4:** Post-intervention comparison of clinical outcomes across study arms (adjusted for baseline FEV₁)

Outcome	Arm 0 (*n* = 33)	Arm 1 (*n* = 31)	Arm 2 (*n* = 33)	Adjusted *p*-value
CAT score	29.88 ± 13.69	24.84 ± 13.69	18.82 ± 13.69	< 0.001
mMRC score	1.36 ± 0.39	1.00 ± 0.39	0.91 ± 0.39	< 0.001
FEV₁ (% predicted, post)	60.27 ± 14.38	68.52 ± 10.38	69.97 ± 10.17	0.001
Moderate exacerbations	2 (1–3)	2 (1–2)	1 (1–2)	0.28
Duration of moderate exacerbation (weeks)	3.23 ± 1.49	2.17 ± 1.49	1.50 ± 1.49	0.033
Severe exacerbations	0 (0–1)	0 (0–1)	0 (0–1)	0.81
SGRQ total score (post)	16.57 ± 1.81	11.86 ± 0.52	10.11 ± 1.10	< 0.001

### Exacerbations outcomes

While Pharmaceutical care exhibited the lowest mean number of severe exacerbations, this difference was not statistically significant compared to the other groups. These findings suggest that neither counselling nor pharmaceutical care interventions significantly impacted the number of severe exacerbations compared to the control group. The mean change differed slightly across the three study groups. The Arm 0 showed the highest mean change (0.63 ± 0.74), followed by the Arm 1 (0.44 ± 0.53) and the Arm 2 (0.20 ± 0.45) (Table 5 (a)). However, pairwise comparisons between groups did not demonstrate statistically significant differences (*p* > 0.05), indicating that the observed changes were comparable across the study arms Table 5 (b).

**Table 5(a):** Severe exacerbations across groups

Study Arm	*n*	Mean ± SD	95% CI (Mean)
Arm 0	33	0.63 ± 0.74	0.09–1.17
Arm 1	31	0.44 ± 0.53	0.05–0.83
Arm 2	33	0.20 ± 0.45	-0.29–0.69

**Table 5(b):** Pairwise comparisons of severe exacerbations

Comparison	Mean Difference	*p*-value
Arm 0 vs. Arm 1	0.181	0.813
Arm 0 vs. Arm 2	0.425	0.447
Arm 1 vs. Arm 2	0.244	0.751

### Disease progression

Changes in GOLD classification between pre- and post-intervention showed that most patients in highest percentage of patients experienced no progression in the Arm 2 (93.9%), followed by the Arm 1 (80.6%) and Arm 0 (60.6%). Progression by one grade was more frequent in Arm 0 (36.4%) compared with the Arm 1 (16.1%) and Arm 2 (3.0%). Only a small proportion of patients showed improvement, observed in the Arm 1 (3.2%) and Arm 2 (3.0%), while no improvement was noted in the Arm 0 (Table 6).

**Table 6:** Disease progression analysis across groups on the bases of spirometry grades

Change category (Post vs. Pre)	Arm 0 *N* (%)	Arm 1 *N* (%)	Arm 2 *N* (%)
Improved	0 (0.0)	1 (3.2)	1 (3.0)
No progression	20 (60.6)	25 (80.6)	31 (93.9)
Progressed by 1 Grade (1→2 or 2→3)	12 (36.4)	5 (16.1)	1 (3.0)
Progressed by ≥ 2 Grades	1 (3.0)	0 (0.0)	0 (0.0)

Spirometry Grades: Grade 1: ≥80%; Grade 2; 50–79%; Grade 3; 30–49%;Grade 4: <30%

### Quality of life (SGRQ)

Quality of life was evaluated using SGRQ. The detailed analysis of SGQR symptoms score, activity score and impact scores is present in supplementary file S1. Table 7. shows the within groups the changes in SGRQ total scores from pre- to post-intervention. Usual care group demonstrated a significant worsening in overall health status, with mean scores increasing from 10.15 ± 2.89 at baseline to 16.57 ± 1.81 post-intervention (mean difference 6.42 ± 0.58; *p* = 0.001). Similarly, educational intervention group showed a statistically significant deterioration in total scores, rising from 9.66 ± 2.23 to 11.86 ± 0.52 (mean difference 2.20 ± 0.50; *p* = 0.001). In contrast, Pharmaceutical Care group exhibited only a minimal increase from 9.76 ± 2.74 to 10.11 ± 1.10 (mean difference 0.35 ± 0.45), which was not statistically significant (*p* = 0.41). Overall, these findings indicate a significant worsening of overall disease-related health status in both usual care and educational intervention group, while no meaningful change was observed in Pharmaceutical care group Table 7.

**Table 7:** SGRQ quality of Life (QoL) scores before and after the intervention across the groups

Comparison	Pre intervention Score (Mean ± SD)	Post intervention Score (Mean ± SD)	Difference in (Mean ± SD)	Std. Error	*P*-value
Arm 0	10.15 ± 2.89	16.57 ± 1.81	6.42 **±** 0.58	0.58	0.001
Arm 1	9.66 ± 2.23	11.86 ± 0.52	2.2 **±** 0.5	0.5	0.001
Arm 2	9.76 ± 2.74	10.11 ± 1.10	0.35 **±** 0.45	0.45	0.41

Note: Arm 0 = Usual Care; Arm 1 = Educational Intervention; Arm 2 = Pharmaceutical Care

## Discussion

This study evaluated the effectiveness of dispensing pharmaceutical care based on the GOLD 2020 recommendations on various clinical outcomes in patients with COPD. Our results indicated that while both pharmacist counselling and comprehensive pharmaceutical care have beneficial impacts on several clinical endpoints, the most pronounced improvements were observed in the pharmaceutical care group. These improvements spanned across QoL, symptom control as CAT and mMRC, preservation of lung function (FEV₁), and slowing down of the disease progression but no effect on number of acute exacerbations and hospital visits. It should be noted that this study used the GOLD 2020 classification framework, as the trial was designed, registered, and conducted during that period. Although recent GOLD updates have modified patient grouping systems, the baseline characteristics of our cohort particularly higher CAT scores, mMRC ratings, and a history of exacerbations suggest that many participants would fall within higher symptom-burden categories in the updated framework. Therefore, the observed benefits of pharmaceutical care are expected to remain clinically relevant under current GOLD recommendations.

This study demonstrates that a structured, guideline-directed approach to COPD management is associated with improvements in symptom control, quality of life, and preservation of lung function. These findings are consistent with previous reports indicating that adherence to GOLD-recommended management strategies leads to better clinical outcomes in COPD patients [49].

The observed improvements can be explained by the integration of multiple evidence-based components, including optimization of pharmacotherapy, regular patient monitoring, and reinforcement of disease-specific education. Similar findings have been reported in earlier studies, where structured interventions and multidisciplinary care models were associated with improved symptom burden and reduced disease progression [50, 51]. In particular, studies evaluating guideline implementation strategies have shown that consistent follow-up and treatment adjustment play a central role in achieving better disease control [52].

The improvement in quality of life observed in this study aligns with prior research demonstrating that patient-centered care approaches, including education and close clinical supervision, contribute significantly to better patient-reported outcomes [53]. Likewise, previous studies have highlighted that proactive management strategies can help slow the decline in lung function, supporting the findings observed in the present study [54].

From a clinical perspective, these results reinforce the importance of implementing structured care pathways in routine practice. Evidence from earlier studies suggests that systematic application of guideline-based management improves overall disease outcomes, regardless of the specific healthcare provider involved [55]. Therefore, the benefits observed in this study are likely attributable to improved adherence to evidence-based care rather than a single intervention component.

## Conclusions

In summary, this study demonstrates that structured implementation of guideline-directed COPD management can lead to meaningful improvements in symptom control, quality of life, and preservation of lung function. While these findings support the value of a more organized and proactive approach to patient care, it is important to interpret them within the context of real-world clinical practice. The observed benefits are likely driven by consistent application of evidence-based recommendations, regular patient engagement, and closer monitoring, rather than any single component of the intervention. At the same time, the feasibility of implementing such approaches on a larger scale will depend on available resources, healthcare infrastructure, and the ability to integrate multidisciplinary care into routine practice. Future studies with larger populations and longer follow-up periods are needed to confirm these findings and to better understand their long-term clinical and economic impact.

### Limitations and addressing potential bias

Despite the promising findings of this study, several limitations must be acknowledged.

1. Sample Size and Study Duration: While the study enrolled 120 participants, only 97 completed the trial. This attrition may have introduced selection bias, as patients who dropped out might have differed systematically from those who completed the study. The number of exacerbation events observed during the study period was relatively small, which may have limited the statistical power to detect differences between study groups for this outcome.
2. Potential Bias in Outcome Assessments: Although the study employed a randomized controlled trial (RCT) design, certain biases remain possible. Performance bias could have arisen due to the nature of the intervention, as participants in the pharmaceutical care group may have been more motivated to adhere to treatments given the additional education and support provided. Detection bias is also a consideration, as patient-reported outcomes, such as the COPD Assessment Test (CAT) and Modified Medical Research Council (mMRC) dyspnea scale, could be influenced by patient perception rather than objective measurements.
3. Imprecision in Measuring Adherence: Medication adherence was not quantitatively measured in this study. Although patient counselling and follow-up were provided, adherence was assessed qualitatively through clinical interviews rather than validated adherence scales.
4. Multiplicity of Analyses: Given the multiple outcomes assessed—including symptom scores, lung function, exacerbation frequency, and quality of life—there is a risk of Type I errors. Although statistical corrections (such as ANOVA and post hoc Tukey’s tests) were applied, additional studies with confirmatory analyses are warranted to ensure the robustness of these findings.
5. Evolution of GOLD Guidelines: The present study followed the GOLD 2020 classification system because the trial design, ethical approvals, and protocol registration were completed during that period. Since then, the GOLD strategy has undergone updates that modified patient grouping frameworks. Although these updates were not applied during the trial analysis, the overall interpretation of results remains applicable because the core principles of symptom assessment and exacerbation risk stratification remain consistent across guideline versions.

### Generalizability and external validity

The external validity of this study is influenced by several factors:

1. Single-Center Design: The study was conducted at a single tertiary care hospital in Pakistan, limiting its generalizability to other healthcare settings, particularly those with different healthcare infrastructures, socioeconomic conditions, and patient demographics. Future multicenter trials across diverse regions would help validate these findings.
2. Healthcare System Variability: The effectiveness of pharmacist-led interventions may depend on healthcare system structures, availability of trained professionals, and patient access to care. The model tested in this study may need adaptation for different settings, particularly in low-resource environments where pharmacist roles in direct patient care may be limited.
3. Cultural and Socioeconomic Factors: Patient beliefs, health literacy, and financial constraints may influence treatment adherence and response to pharmaceutical care interventions. While this study demonstrated significant improvements in clinical outcomes, the impact of similar interventions in high-income or culturally distinct populations remains to be explored.

## Supplementary Information

Below is the link to the electronic supplementary material.

Supplementary Material 1

## Data Availability

The datasets used and/or analysed during the current study are available from the corresponding author on reasonable request.

## Declarations

### Clinical trial protocols

The study protocols are the detailed version of the approved clinical trial that has been published in **Heliyon **10.1016/j.heliyon.2023.e21539 [48].
