# Supplementary material for: Management of COPD and comorbidities in COPD patients by dispensing pharmaceutical care following Global Initiative for Chronic Obstructive Lung Disease-guidelines (GOLD guidelines 2020): a prospective randomized clinical trial
Source: BMC Health Serv Res. 2026 Jun 16;26:1184. doi: 10.1186/s12913-026-14802-w (PMC13508290; doi:10.1186/s12913-026-14802-w)
Supplement: Supplementary file 1 [file 12913_2026_14802_MOESM1_ESM.docx]

**Supplementary File S1**

# Detailed Intervention Protocols

This supplementary file provides a detailed description of the intervention protocols used in the randomized clinical trial evaluating pharmacist-led pharmaceutical care for COPD management. The interventions were designed according to the Global Initiative for Chronic Obstructive Lung Disease (GOLD 2020) recommendations to ensure standardized delivery across all study arms.

The supplementary material outlines the procedures followed during intervention delivery, patient monitoring, and outcome assessment. A comparison of interventions implemented in the three study arms is also presented.

## 1. Study Arms Overview

Participants enrolled in the study were randomly assigned to one of three arms in a 1:1:1 ratio:

- Arm 0 – Usual Care (Control Group): Participants received routine physician‑directed COPD management.
- Arm 1 – Educational Intervention: Participants received usual care plus structured pharmacist‑led patient education (Table S1).
- Arm 2 – Pharmaceutical Care Intervention: Participants received educational intervention plus structured pharmacist‑led pharmaceutical care (Table S1 & S2).

**Table S1. Overview of the interventions provided to the study group**

| **Intervention Component** | **Arm 0** | **Arm 1** | **Arm 2** |
| --- | --- | --- | --- |
| **Routine physician care** | ✓ | ✓ | ✓ |
| **COPD patient education** | — | ✓ | ✓ |
| **Medication counseling** | — | ✓ | ✓ |
| **Inhaler technique training** | — | ✓ | ✓ |
| **Lifestyle counseling** | — | ✓ | ✓ |
| **Pharmacotherapy review** | — | — | ✓ |
| **Medication optimization** | — | — | ✓ |
| **Symptom monitoring (CAT/mMRC)** | — | — | ✓ |
| **Exacerbation monitoring** | — | — | ✓ |
| **Medication adherence monitoring** | — | Limited | ✓ |
| **Comorbidity monitoring** | — | — | ✓ |
| **Vaccination counseling** | — | — | ✓ |
| **Structured follow‑up** | — | Limited | ✓ |

**Table S2. Summary of Pharmacotherapy Optimization Interventions in Arm 2 (Pharmaceutical Care Group)**

| **Therapeutic intervention / medication optimization** | **Number of participants (%) (n=33)** |
| --- | --- |
| Escalation from short-acting bronchodilator only to LABA or LAMA maintenance therapy | 11 (33.3%) |
| Addition of dual bronchodilator therapy (LABA + LAMA) | 9 (27.3%) |
| Addition of inhaled corticosteroid (ICS)-containing regimen | 6 (18.2%) |
| De-escalation of inappropriate ICS-containing therapy | 4 (12.1%) |
| Inhaler device technique correction/training | 28 (84.8%) |
| Medication adherence reinforcement | 30 (90.9%) |
| Optimization of comorbidity-related pharmacotherapy | 14 (42.4%) |
| Smoking cessation counseling/pharmacologic advice | 18 (54.5%) |
| Vaccination recommendation (influenza/pneumococcal) | 10 (30.3%) |

**Footnote:** Percentages are calculated based on participants allocated to Arm 2 (Pharmaceutical Care). Multiple interventions could be applied to the same participant.

## Figure S1. Intervention Workflow Diagram

A pictorial flowchart illustrating the intervention workflow and monitoring process across the study arms is presented in Figure S1.

**Figure S1.** Flowchart illustrating the intervention protocols used in the randomized clinical trial. Participants were randomized into three study arms: usual care (Arm 0), educational intervention (Arm 1), and pharmacist-led pharmaceutical care (Arm 2). Educational and pharmaceutical interventions were delivered by the pharmacist, while clinical outcomes including CAT score, mMRC dyspnea scale, lung function, exacerbations, and quality of life were assessed during follow-up.

##### **SGRQ symptoms scores of the groups (Pre and Post- Intervention)**

**Table S3**. presents the within-group changes in SGRQ Symptoms scores from pre- to post-intervention. Usual care demonstrated a marked **worsening of symptoms**, with mean scores increasing from **47.99 ± 5.18** at baseline to **78.35 ± 8.45** post-intervention (mean difference **30.36 ± 9.91**), and this change was statistically significant (**p < 0.001**). Similarly, educational intervention showed a **significant deterioration in symptom scores**, increasing from **46.76 ± 2.52** to **57.40 ± 3.09** (mean difference **10.64 ± 3.99; p < 0.001**). In contrast, Pharmaceutical care exhibited only a small increase in symptom scores from **45.97 ± 5.60** to **47.60 ± 5.80**, which was **not statistically significant (p = 0.254**). Overall, these findings indicate a significant worsening of symptom burden in Groups A and B, while symptom severity remained relatively stable in Pharmaceutical care group.

**Table S3. SGRQ symptoms scores among the groups.**

|  | **Pre intervention score (Mean ± SD)** | **Post intervention score (Mean ± SD)** | **Difference in (Mean ± SD)** | **Standard Error** | **P-value** |
| --- | --- | --- | --- | --- | --- |
| **Arm 0** | 47.99 ± 5.18 | 78.35 ± 8.45 | 30.36 ± 9.91 | 1.73 | < 0.001 |
| **Arm 1** | 46.76 ± 2.52 | 57.40 ± 3.09 | 10.64 ± 3.99 | 0.72 | < 0.001 |
| **Arm 2** | 45.97 ± 5.60 | 47.60 ± 5.80 | 1.63 ± 8.06 | 1.40 | 0.254 |

Note: Symptoms scores from pre-intervention to post-intervention within each group were tested using the paired samples t-test.”

##### **Activity scores SGRQ activity scores of the groups**

**Table S4.** presents the within-group changes in **SGRQ Activity** scores from pre- to post-intervention. Usual care group showed a substantial **worsening in activity limitation**, with mean scores increasing from **24.48 ± 9.28** at baseline to **39.97 ± 15.15** post-intervention (mean difference **15.49 ± 17.77**), and this change was statistically significant (**p < 0.001**). Educational intervention group also demonstrated a **significant deterioration** in activity scores, rising from **22.68 ± 6.49** to **27.84 ± 7.97** (mean difference **5.16 ± 10.28; p = 0.009**). In contrast, Pharmaceutical care exhibited only a minimal increase from **21.35 ± 12.28** to **22.11 ± 12.71** (mean difference **0.76 ± 17.67**), which was **not statistically significant (p = 0.806**). Overall, these findings suggest significantly increased activity-related impairment in usual care and educational interventions group, while Pharmaceutical care remained quite stable.

Table S4. SGRQ Activity scores among the groups.

| **Comparison** | **Pre intervention score (Mean ± SD)** | **Post intervention score (Mean ± SD)** | **Difference in (Mean ± SD)** | **Standard Error** | **P-value** |
| --- | --- | --- | --- | --- | --- |
| **Arm 0** | 24.48 ± 9.28 | 39.97 ± 15.15 | 15.49 ± 17.77 | 3.09 | < 0.001 |
| **Arm 1** | 22.68 ± 6.49 | 27.84 ± 7.97 | 5.16 ± 10.28 | 1.85 | 0.009 |
| **Arm 2** | 21.35 ± 12.28 | 22.11 ± 12.71 | 0.76 ± 17.67 | 3.08 | 0.806 |

Note: Activity scores from pre-intervention to post-intervention within each group were tested using the paired samples t-test.”

**SGRQ impact scores of the groups**

**Table S5** presents the within-group changes in **SGRQ impact** scores from pre- to post-intervention. Usual care group demonstrated a **marked worsening of overall disease impact**, with mean scores increasing from **3.22 ± 0.75** at baseline to **5.26 ± 1.22** post-intervention (mean difference **2.04 ± 1.43**), and this change was statistically significant (**p < 0.001**). Educational intervention group also showed a **significant deterioration in impact scores**, rising from **2.70 ± 0.07** to **3.31 ± 0.08** (mean difference **0.61 ± 0.11; p < 0.001**). In contrast, Pharmaceutical care exhibited only a minimal increase from **3.16 ± 0.48** to **3.27 ± 0.50** (mean difference **0.11 ± 0.69**), which was **not statistically significant (p = 0.369**). Overall, these findings indicate that the impact remained relatively stable in Pharmaceutical care.

**Table S5** SGRQ impact scores among the groups.

| **Comparison** | **Pre intervention score (Mean ± SD)** | **Post intervention score (Mean ± SD)** | **Difference in (Mean ± SD)** | **Standard Error** | **P-value** |
| --- | --- | --- | --- | --- | --- |
| **Arm 0** | 3.22 ± 0.75 | 5.26 ± 1.22 | 2.04 ± 1.43 | 0.25 | < 0.001 |
| **Arm 1** | 2.70 ± 0.07 | 3.31 ± 0.08 | 0.61 ± 0.11 | 0.02 | < 0.001 |
| **Arm 2** | 3.16 ± 0.48 | 3.27 ± 0.50 | 0.11 ± 0.69 | 0.12 | 0.369 |

**Note:** Impact scores from pre-intervention to post-intervention within each group were tested using the paired samples t-test.”
